# Trends in the prevalence of social isolation among middle and older adults in China from 2011 to 2018: the China Health and Retirement Longitudinal Study

**DOI:** 10.1186/s12889-024-17734-4

**Published:** 2024-02-01

**Authors:** Yanwei Lin, Tingting Zhu, Xiaoyan Zhang, Zhirong Zeng

**Affiliations:** 1 Department of Social Medicine and Health Management, School of Public Health, Guangdong Medical University, Dongguan, China; 2 Department of Epidemiology and Health Statistics, School of Public Health, Guangdong Medical University, Dongguan, China; 3 Institute of Public Health and Wellness, Guangdong Medical University, 1#, Xincheng Avenue, Songshanhu District, Dongguan, Guangdong 523808 China; 4 Care Policy and Evaluation Centre (CPEC), London School of Economics and Political Science, London, WC2A2AE UK

**Keywords:** Social isolation, Trend, Middle-aged and older adults, Longitudinal study, China

## Abstract

**Background:**

Epidemiological studies have shown that social isolation, which is prevalent in older adults, is associated with a range of adverse health outcomes, but the prevalence of and trends in regard to social isolation remain ambiguous in China. The aim of this study was to elucidate the trends regarding the prevalence of social isolation among middle-aged and older adults in China from 2011 to 2018 and to further identify associated risk factors.

**Methods:**

A repeated cross-sectional study, The data were derived from panel sample data of four waves conducted from May 2011 to August 2018 in the nationally representative China Health and Retirement Longitudinal Study (CHARLS) using multistage probability sampling. Social isolation was ascertained by the five item Steptoe Social Isolation Index. The potential covariates were demographic characteristics, lifestyle factors, and health status. Linear-by-linear association was used to assess the trends in regard to social isolation over time under the influence of the potential covariates. Linear-by-linear association and an age-period-cohort analysis were used to explore the trends, and two-level (time, individual) generalized estimating equation models (GEE) linked multivariate binary logistic regression were performed to identify risk factors.

**Results:**

A high prevalence of social isolation and a moderate upward trend from 2013 to 2018 were observed among a U-shaped trend prevalence of social isolation from 2011 to 2018 across China, with rates of 38.09% (95% CI = 36.73–39.45) in 2011, 33.66% (32.32–35.00) in 2013, 39.13% (37.59–40.67) in 2015, and 39.95% (38.59–41.31) in 2018 (*p* < 0.001). The prevalence of social isolation increased with age and educational attainment. Females had a higher prevalence than males. The prevalence of social isolation was found to be significantly lower in pensioners than in non-pensioners between 2011 and 2018 (*p* < 0.001). The prevalence of social isolation was 38.9%, 34.9%, 38.5%, and 44.08% about three times higher among those who doid not use the Internet and 13.44%, 11.64%, 12.93%, and 16.73% than among those who doid in 2011, 2013, 2015 and 2018 respectively. The participants with short (0–5 h) and long sleep (9 or more hours), and poor self-rated health had a higher prevalence of social isolation than the others. Older age, lower educational attainment, living in a rural region, lack of medical insurance or pension, lack of internet use and poor health were risk factors (*p* < 0.05).

**Conclusions:**

We found a U-shaped prevalence of social isolation trends from 2011 to 2018 and revealed increasing trends from 2013 to 2018 among middle-aged and older adults in China. The findings of the study highlight the urgent need for interventions to reduce social isolation including improving sleep quality and internet skills. Disadvantaged groups in terms of age, economic status, and health status should be the focus of such interventions, especially in the era of COVID-19.

**Supplementary Information:**

The online version contains supplementary material available at 10.1186/s12889-024-17734-4.

## Background

Social isolation is defined as ‘‘a state in which the individual lacks a sense of social belonging, lacks engagement with others, has a minimal number of social contacts and lacks fulfilling and quality relationships’’ [1]. It has been identified and well-documented by previous research as a social determinant of physical and mental health. Particularly for older adults living in the community, social isolation leads to numerous detrimental health effects, including not only higher rates of morbidity and mortality, but also dementia and an increased rate of falls. Though a number of studies have confirmed that social distancing can slow the rate of transmission of COVD-19 [2, 3], it also brings about health problems such as depression, anxiety and stress [4]. A German study found that the prevalence of social isolation was 12.3% across ages 18–79 years, and increased with increasing age, from 5.4% in the 18-39 age group to 21.7% in the 70-79 age group [5]. A survey in the United States reported that 35% of adults aged 45 and over felt socially isolated [6]. A systematic review showed that the prevalence of social isolation among older adults was 31.2% during the coronavirus disease 2019 (COVID-19) pandemic [7]. Overall, social isolation has a high prevalence in older age groups and is strongly associated with poor health conditions and unfavorable behaviors [8]. Undoubtedly, social isolation has become a major health issue for older adults living in the community, but the epidemiological data on social isolation have been inconsistent. While there has been an ongoing debate about the rise of social isolation based on reports of low birth rates, an ageing population and so on, but there is little evidence of rising rates of social isolation. As social networks shrink with age, with a marked increase in the proportion of older adults, it has been hypothesised that social isolation may indeed increase over time [5]. However, there have been relatively few reports on the changes in the prevalence of social isolation among older adults at the population level in recent years, especially in China [9].

Previous studies have demonstrated that the causes of social isolation are complicated. The atrophy of older adults’ social networks occurs due to factors specific to the individual, such as a decline in physical or cognitive function associated with ageing [10], and social distancing as recommended due to the COVID-19 pandemic [11]. In addition, barriers to internet access among middle-aged and older adults may exacerbate their social isolation [12]. As a result of these individual and environmental factors, the social networks of middle-aged and older adults gradually decline. A higher risk of social isolation is observed among older adults, and more than 50% of this population category are at risk of social isolation globally [13]. However, studies on social isolation trends have focused largely on loneliness and refer mainly to the frequency of social engagement or number of confidants. For example, studies have found that the mean network size decreased by about a third (one confidant), from 2.94 in 1985 to 2.08 in 2004 [14], and social isolation increased from 2003 to 2020. In particular, social engagement with family, friends and others (roommates, neighbors, acquaintances, co-workers, clients, etc.) decreased among Americans [15]. A repeated cross-sectional comparative study in Japan and England found that social isolation among older adults increased from 2010 to 2016 in Japan, which was a more severe increase than in England, where social isolation was gradually alleviated among women aged 75 and older over the same period [13]. In summary, we find that studies on trends in social isolation are not only limited and mainly focused on the United States, but also vary from country to country. However, there is a consensus that social isolation has not received adequate attention in public health, nor has its extent and trends at the national and subgroup levels been paid much attention.

The indicators used to assess social isolation vary widely and often include living alone, being unmarried, low participation in social activities and a lack of diversity in social networks [6], which is related to the uncertainty and incomparability of the social isolation prevalence proxy. The adverse health effects of social isolation are increasingly being recognized. In addition to the increased risk of poor physical health due to cardiovascular disease [16], premature death [17], and functional impairment [18], social isolation hinders social integration and potentially triggers mental health problems, such as depression and anxiety disorders [19, 20]. Age [21, 22], residential area [23] and educational attainment [24] are known risk factors for social isolation among demographic characteristics. Sleep problems [25] and internet use [26, 27] also have an impact on social isolation. Poor self-rated health [28], a low capacity for activities of daily living [29] and multiple chronic diseases [30] have also been documented. In addition, family characteristics [31], living arrangements [32] and children [33] might be factors. However, studies are inconclusive about the differences and risk factors in the prevalence of social isolation in relation to socio-demographic and social factors [5].

In 2020, China’s Seventh Census reported that there were 264 million Chinese adults who were more than 60 years old, representing 18.70% of the population. This percentage is expected to rise to 26.9% by 2050 [34, 35]. In parallel with the ageing population, social isolation, a state of low participation in social activities, and poor integration into social life [36] are prevalent among older adults as they experience significant life changes, including retirement, death of, or separation from family members and friends, and a decreased ability to perform activities of daily living, which has become a significant public health issue of global concern [37]. The World Health Organization’s (WHO’s) Active Ageing Initiative recognized the significance of reducing social isolation as early as 2002 [38], and it has been included as one of the four key areas for action in the WHO Healthy Ageing Ten Year Strategy (2021–2030) [39]. Cultural tradition emphasizes the importance of family and social networks in Chinese populations. It has been suggested that the association between social isolation and health may be more pronounced. However, very few studies have examined the prevalence of social isolation among middle-aged and older Chinese adults [9], and even fewer have examined the changes in its trends over time. The trends and social risk factors regarding social isolation remain ambiguous in China.

Given the dramatic changes in the use of technology as a means of social connectivity in China, it is currently not well understood whether such changes will exacerbate the trend of social isolation. This study aims to elucidate the trends regarding the prevalence of social isolation among middle-aged and older adults in China from 2011 to 2018 and to further identify the associated risk factors. The study included adults aged 45 years and older, as social functional decline can begin at this age. We hypothesised that social isolation may have worsened as the technological and economic development of society has accelerated, and our findings contribute to the growing body of literature focusing on social isolation and provide important information for the ageing population.

## Methods

### Data and sampling

Our study is a repeated cross-sectional study. The data were derived from the nationally representative China Health and Retirement Longitudinal Study (CHARLS), conducted by the National Development Institute of Peking University [40]. The CHARLS has been conducted four times since 2011 to assess the economic, social, and health conditions of adults aged 45 years and older in China. Stratified multistage sampling was conducted according to a probability proportionate to size [41]. A total of 150 counties in 28 provinces across China were included in the final sample. The CHARLS survey was approved by the Peking University Biomedical Ethics Economic Review Committee (IRB00001052-11015), and all of the participants provided written informed consent. A detailed description of the CHARLS has been published elsewhere [42].

**Fig. 1:**
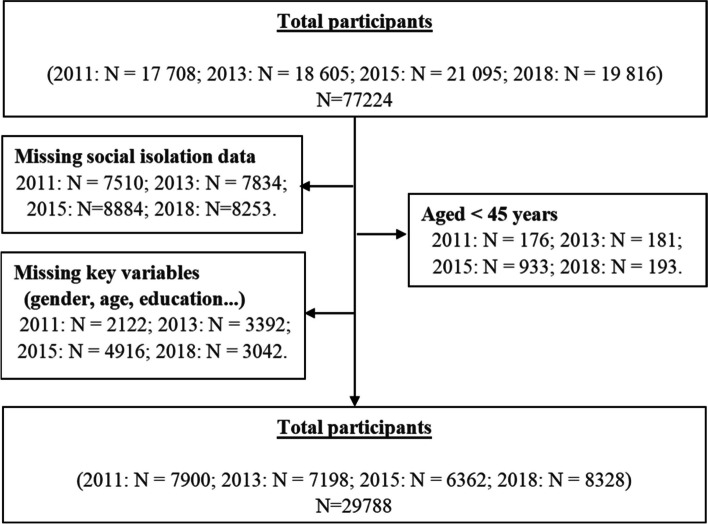
Flowchart for selection of the final sample included in the analysis

To examine the trends in the prevalence of social isolation among middle-aged and older people in different years, panel data of four waves was chosen for this study from 2011 to 2018. The sample was selected from the CHARLS according to the following eligibility criteria: 1) the participants were aged 45 years or older and 2) the participants had answered all of the questions on the assessment of social isolation and its covariates. Referring to previous studies [43], the study sample was limited to participants who provided information on all indicators of social isolation. After removing participants with more than 20% missing items for social isolation, Little's MCAR tests were performed on the remaining missing values for social isolation at each wave, and none were significant, suggesting that the complete random missing condition was met and could be removed directly. In addition, for the hierarchical linear analyses, we further restricted the participants to those who provided complete information on all covariates [43]. A total of 29788 participants were included in the final analyses. Figure 1 shows the details of the participant selection.

### Measurements

#### Social isolation

In line with previous studies [44, 45], social isolation was ascertained using five-items similar to the Steptoe Social Isolation Index, but with slight differences [46], which asks about marital/cohabitation status, monthly contact (face-to-face, telephone and email/written) with children, and other family or friends, and participation in groups. These are the five items: 1) whether the participant was cohabiting; 2) whether the participant or his/her spouse had seen their parent or parent-in-law in the past month; 3) whether the participant had seen or contacted his/her child in the past month (including by phone, text message, post or e-mail); 4) whether the participant had interacted with his/her friends in the past month; and 5) whether the participant had participated in any activities (such as social clubs or residents’ groups, religious groups, or committees) in the past month. For the above five questions, if the answer was no, 1 point was allocated, otherwise 0 points were allocated, resulting in a total social isolation score ranging from 0 to 5. Based on the total score, participants were divided into non-isolated (score = 0–2) and isolated groups (score = 3–5), based on previous studies [47].

#### Covariates

The potential covariates were demographic characteristics (age, gender, educational attainment, residence, geographic location, medical insurance and pension), lifestyle factors (internet use, sleep duration, smoking and alcohol consumption), and health status (self-rated health, multimorbidity, and physical and mental health). Physical health was measured using Katz’s Basic Activities of Daily Living scale (BADL, Cronbach’s α = 0.881–0.889), which includes the following six items: the ability to bathe, eat, get in and out of bed, get dressed, go to the toilet, and defecate. The BADL scores were classified into three levels of impairment based on previous studies: 1) none: BADL score = 0, 2) mild: BADL score = 1, and 3) severe: BADL score = 2 [48–50]. Mental health was assessed using the 10-item Epidemiological Survey Center Depression Scale Short Form (CES-D-10 scale, Cronbach’s α = 0.78–0.79), which consists of 10 items, with scores ranging from 0 to 30. The scores are divided into three levels: 1) depression: score ≥ 20, 2) depressive symptoms: score 10–19, and 3) no depressive symptoms: score < 10) [51, 52]. Details of the scoring systems are presented in Table S1.

### Statistical analysis

For descriptive statistics, frequencies and percentages were used for the categorical variables, and the means and standard deviations were used for the continuous variables. To eliminate the effect of differences in the internal composition of the cross-sectional data in different years on the prevalence rate, we adopted sampling weights to account for differences in sampling fractions in the panel data compared to the overall population. using direct standardization in regard to the population stratum proportions of sex. Sex-standardized rate that is a statistical adjustment applied to rates or ratios to account for differences in the distribution of a particular characteristic was calculated. A chi-squared test was used to examine the differences in the prevalence of social isolation between the subgroups according to each categorical variable. Linear-by-linear association was used to assess the trends in social isolation over time under the influence of confounding variables. To separate the layer effects over time and minimize the influence of reverse causality, two-level (time, individual) generalized estimating equation models (GEE) linked multivariate binary logistic regression were performed to test the trend difference between the subgroups, and risk factors were identified. An age-period-cohort analysis (APC) was performed to test the effects of the participant age, the survey year, and birth cohort on the prevalence of social isolation. To minimize the expected loss relative to the appropriate reference posterior distribution, the Intrinsic Estimator was used to independently estimate the effect coefficients of age, survey period, and birth cohort [53]. Stata 15.0 (StataCorp, College Station, TX, USA) and SPSS 25.0 (IBM, Armonk, NY, USA) was used to perform the data analysis. The significance level was set at a two-tailed value of *p* < 0.05.

## Results

### Participants’ characteristics

The total sample consisted of 29,788 participants, including 7,900 in 2011, 7,198 in 2013, 6,362 in 2015, and 8,382 in 2018. The mean (standard deviation) age was 60.4 (9.61) years, and the ratio of men to women was approximately 1:1 (49.1%: 50.9%). The majority (62.3%) of the participants had a primary education or less, and 17,706 (59.4%) lived in rural areas. Almost all of the participants (94.8%) had health insurance, and 19,057 (64.0%) had a pension. Surprisingly, the majority of the participants (92.3%) had not used the internet in the past month, and more than half (54.6%) of the participants slept less than 7 hours per night. In addition, 9,422 (31.6%) smoked, and 10,576 (35.5%) consumed alcohol. 13,599 (45.7%) participants had multimorbidity, 7,259 (24.4%) reported poor health, 11,133 (37.4.%) had symptoms of depression, and 2,187 (7.3%) had a severe physical impairment. The characteristics of the samples from the four waves of data are shown in Table 1.

**Table 1:** Characteristics of the participants classified as middle-aged or older adults (*N* = 29 788)^a^

	Participants^b^									*P * value^d^
	Overall (*N* = 29 788)	Year^c^								
		2011		2013		2015		2018		
		2011 (*n* = 7900)	social isolation scores ≥ 3^e^	2013 (*n* = 7198)	social isolation scores ≥ 3^e^	2015 (*n* = 6362)	social isolation scores ≥ 3^e^	2018 (*n* = 8328)	social isolation scores ≥ 3^e^	
**Sociodemographic characteristics**										
Gender										
Male	15159 (50.9)	4024 (50.9)	1513 (50.3)	3639 (50.6)	1213 (50.1)	3357 (52.8)	1241 (50.0)	4139 (49.7)	1615 (48.5)	0.003
Female	14629 (49.1)	3876 (49.1)	1496 (49.7)	3559 (49.4)	1210 (49.9)	3005 (47.2)	1243 (50.0)	4189 (50.3)	1714 (51.5)	
Age (years)										
45 ~	14753 (49.5)	4408 (55.8)	1112 (37.0)	3722 (51.7)	776 (32.0)	2904 (45.6)	677 (27.3)	3719 (44.7)	868 (26.1)	< 0.001
60 ~	9543 (32.0)	2233 (28.3)	1088 (36.2)	2291 (31.8)	950 (39.2)	2192 (34.5)	992 (39.9)	2827 (33.9)	1318 (39.6)	
70 ~	4504 (15.1)	1035 (13.1)	634 (21.1)	981 (13.6)	555 (22.9)	1030 (16.2)	634 (25.5)	1458 (17.5)	907 (27.2)	
80 ~	988 (3.3)	224 (2.8)	175 (5.8)	204 (2.8)	142 (5.9)	236 (3.7)	181 (7.3)	324 (3.9)	236 (7.1)	
Educational attainment										
Primary school or below	18557 (62.3)	5035 (63.7)	2311 (76.8)	4482 (62.3)	1863 (76.9)	3902 (61.3)	1856 (74.7)	5138 (61.7)	2405 (72.2)	0.002
Middle and high school	10466 (35.1)	2634 (33.3)	658 (21.9)	2553 (35.5)	534 (22.0)	2292 (36.0)	591 (23.8)	2987 (35.9)	891 (26.8)	
College and above	765 (2.6)	231 (2.9)	40 (1.3)	163 (2.3)	26 (1.1)	168 (2.6)	37 (1.5)	203 (2.4)	33 (1.0)	
Residence										
Rural	17706 (59.4)	4632 (58.6)	1895 (63.0)	4274 (59.4)	1573 (64.9)	3869 (60.8)	1638 (65.9)	4931 (59.2)	2151 (64.6)	0.06
Urban	12082 (40.6)	3268 (41.4)	1114 (37.0)	2924 (40.6)	850 (35.1)	2493 (39.2)	846 (34.1)	3397 (40.8)	1178 (35.4)	
Geographical location										
East	11003 (36.9)	2976 (37.7)	1049 (34.9)	2689 (37.4)	893 (36.9)	2374 (37.3)	874 (35.2)	2964 (35.6)	1158 (34.8)	0.06
Central	9264 (31.1)	2451 (31.0)	863 (28.7)	2234 (31.0)	704 (29.1)	1918 (30.1)	682 (27.5)	2661 (32.0)	1008 (30.3)	
West	9521 (32.0)	2473 (31.3)	1097 (36.5)	2275 (31.6)	826 (34.1)	2070 (32.5)	928 (37.4)	2703 (32.5)	1163 (34.9)	
Medical insurance										
Yes	28232 (94.8)	7382 (93.4)	2747 (91.3)	6964 (96.7)	2318 (95.7)	5773 (90.7)	2182 (87.8)	8113 (97.4)	3210 (96.4)	< 0.001
No	1556 (5.2)	518 (6.6)	262 (8.7)	234 (3.3)	105 (4.3)	589 (9.3)	302 (12.2)	215 (2.6)	119 (3.6)	
Pension(income)										
Yes	19057 (64.0)	830 (10.5)	352 (11.7)	5879 (81.7)	2019 (83.3)	4856 (76.3)	1863 (75.0)	7492 (90.0)	2950 (88.6)	< 0.001
No	10731 (36.0)	7070 (89.5)	2657 (88.3)	1319 (18.3)	404 (16.7)	1506 (23.7)	621 (25.0)	836 (10.0)	379 (11.4)	
**Lifestyle variables**										
Internet use										
Yes	2298 (7.7)	253 (3.2)	34 (1.1)	381 (5.3)	44 (1.8)	417 (6.6)	53 (2.1)	1247 (15.0)	207 (6.2)	< 0.001
No	27490 (92.3)	7647 (96.8)	2975 (98.9)	6817 (94.7)	2379 (98.2)	5945 (93.4)	2431 (97.9)	7081 (85.0)	3122 (93.8)	
Sleep duration										
0 ~ 5 h	5105 (17.1)	1286 (16.3)	641 (21.3)	1187 (16.5)	510 (21.0)	1064 (16.7)	512 (20.6)	1568 (18.8)	782 (23.5)	< 0.001
5 ~ 7 h	11162 (37.5)	2730 (34.6)	1016 (33.8)	2879 (40.0)	897 (37.0)	2278 (35.8)	833 (33.5)	3275 (39.3)	1215 (39.3)	
7 ~ 8 h	5544 (18.6)	1549 (19.6)	472 (15.7)	1435 (19.9)	405 (16.7)	1132 (17.8)	370 (14.9)	1428 (17.1)	489 (14.7)	
8 ~ 9 h	5770 (19.4)	1710 (21.6)	612 (21.6)	1254 (17.4)	413 (17.0)	1343 (21.1)	503 (20.2)	1463 (17.6)	582 (17.6)	
9 ~ h	2207 (7.4)	625 (7.9)	268 (8.9)	443 (6.2)	198 (8.2)	545 (8.6)	266 (10.7)	594 (7.1)	261 (7.8)	
Smoking										
Yes	9422 (31.6)	2527 (32.0)	959 (31.9)	2606 (36.2)	850 (35.1)	1878 (29.5)	681 (27.4)	2411 (29.0)	938 (28.2)	< 0.001
No	20366 (68.4)	5373 (68.0)	2050 (68.1)	4592 (63.8)	1573 (64.9)	4484 (70.5)	1803 (72.6)	5917 (71.0)	2391 (71.8)	
Alcohol consumption										
Yes	10576 (35.5)	2691 (34.1)	895 (29.7)	2598 (36.1)	748 (30.9)	2393 (37.6)	785 (31.6)	2894 (34.8)	984 (29.6)	< 0.001
No	19212 (64.5)	5209 (65.9)	2114 (70.3)	4600 (63.9)	1675 (69.1)	3969 (62.4)	1699 (68.4)	5434 (65.2)	2345 (70.4)	
**Health status variables**										
Multimorbidity										
Yes	13599 (45.7)	2989 (37.8)	1195 (39.7)	2993 (41.6)	1048 (43.3)	2828 (44.5)	1169 (47.1)	4789 (57.5)	2039 (38.8)	< 0.001
No	16189 (54.3)	4911 (62.2)	1814 (60.3)	4205 (58.4)	1375 (56.7)	3534 (55.5)	1315 (52.9)	3539 (42.5)	1290 (61.2)	
Self-rated health										
Good	7348 (24.7)	1893 (24.0)	578 (19.2)	1789 (24.9)	535 (22.1)	1524 (24.0)	515 (20.7)	2142 (25.7)	695 (20.9)	< 0.001
Fair	15181 (51.0)	3938 (49.8)	1445 (48.0)	3840 (53.3)	1253 (51.7)	3376 (53.1)	1283 (51.7)	4027 (48.4)	1563 (47.0)	
Poor	7259 (24.4)	2069 (26.2)	986 (32.8)	1569 (21.8)	635 (26.2)	1462 (23.0)	686 (27.6)	2159 (25.9)	1071 (32.2)	
Depression^f^										
No depressive symptoms	17214 (57.8)	4048 (51.2)	1334 (44.3)	4812 (66.9)	1517 (62.6)	3927 (61.7)	1476 (59.4)	4427 (53.2)	1678 (50.4)	< 0.001
Depressive symptoms	11133 (37.4)	3479 (44.0)	1468 (48.8)	2189 (30.4)	814 (33.6)	2131 (33.5)	841 (33.9)	3334 (40.0)	1345 (40.4)	
Depression	1441 (4.8)	373 (4.7)	207 (6.9)	197 (2.7)	92 (3.8)	304 (4.8)	167 (6.7)	567 (6.8)	306 (9.2)	
BADL disability^g^										
None	24882 (83.5)	6683 (84.6)	2335 (77.6)	6100 (84.7)	1914 (79.0)	5191 (81.6)	1889 (76.0)	6908 (82.9)	2546 (76.5)	< 0.001
Mild	2719 (9.1)	656 (8.3)	324 (10.8)	649 (9.0)	267 (11.0)	647 (10.2)	305 (12.3)	767 (9.2)	362 (10.9)	
Severe	2187 (7.3)	561 (7.1)	350 (11.6)	449 (6.2)	242 (10.0)	524 (8.2)	290 (11.7)	653 (7.8)	421 (12.6)	

*Abbreviation*: *BADL* basic activities of daily living

^a^Data were collected from 2011 to 2018

^b^Data are presented as the number (percentage) of participants, unless otherwise indicated

^c^Details of the participants in each year’s survey are presented as the number (percentage)

^d^Chi-square test was used to examine the differences between participants with different characteristics

^e^Adapted the prevalence of social isolation index score; scores ranged from 0 to 5. If three or more answers to these questions had responses of “no,” the participant was categorized as “isolated”; otherwise, they were categorized as “non-isolated”

^f^Centre for Epidemiological Studies Depression Scale

^g^Katz’s basic activities of daily living scale

### Trends in social isolation over time

A high prevalence of social isolation and a moderate upward trend from 2013 to 2018 were observed in a U-shaped trend prevalence of social isolation from 2011 to 2018 across China, with rates of 38.09% (95% CI = 36.73-39.45) in 2011, 33.66% (32.32-35.00) in 2013, 39.13% (37.59-40.67) in 2015, and 39.95% (38.59-41.31) in 2018 (*p* < 0.001).

With the exception of age and geographical location, the prevalence of social isolation showed U-shaped trends in the sub-group of the remaining socio-demographic variables, with an upward trend after a low point in 2013. It is worth noting that the prevalence of social isolation increased with age and educational attainment. The prevalence of social isolation was three to four times higher in the over-80s group than in the 45-60s group, and two to three times higher in the group with a primary school education or less than in the group with a college education or more. Females had a higher prevalence than males. In addition, the prevalence of social isolation was significantly higher in rural areas than in urban areas, and among those without health insurance or a pension than among those with health insurance or a pension, suggesting that socio-economically disadvantaged groups were a priority group for social isolation.

Similarly, the prevalence of social isolation presented a U-shaped trend from 2011 to 2018 for most lifestyle and health status subgroups, with an upward trend from 2013 to 2018. The prevalence of social isolation was almost three times higher among those who had not used the internet in the last month than among those who had and it increased significantly over time, as it did among those who slept 7-8 hours and 8-9 hours, but the prevalence of social isolation was significantly higher among those who slept less (0-5 hours) and more (9 or more hours), especially among those who slept the most. Interestingly, there was no increasing trend in the prevalence of social isolation among participants who smoked and consumed alcohol, and the prevalence of social isolation was significantly lower in the group who consumed alcohol than among those who did not, possibly because smoking and drinking alcohol are two forms of social interaction in China, and they may facilitate social networking. In the analyses of health outcomes, the prevalence of social isolation was significantly higher among the subgroup with a poor health status than among those with a good health status and was more likely to increase among participants with multimorbidity and fair or poor self-rated health than among others. Notably, although the prevalence of social isolation was high among participants with physical and mental health problems, as expected, it did not change significantly over the study period (Table 2).

**Table 2:** Standardised prevalence of social isolation in relation to sociodemographic characteristics, lifestyle factors, and health outcomes in middle-aged and older adults in China, 2011–2018

	2011 (*n* = 7900)		2013 (*n* = 7198)		2015 (*n* = 6362)		2018 (*n* = 8328)		
	Overall	the prevalence of social isolation(CI)^a^	Overall	the prevalence of social isolation(CI)^a^	Overall	the prevalence of social isolation(CI)^a^	Overall	the prevalence of social isolation(CI)^a^	*p* value^b^
Year	7900	38.09 (36.73–39.45)	7198	33.66(32.32–35.00)	6362	39.13(37.59–40.67)	8328	39.95(38.59–41.31)	< 0.001
**Sociodemographic subgroups**									
Gender									
Male	4024	1513 (37.6)	3639	1213 (33.3)	3357	1241 (37.0)	4139	1615 (39.0)	0.01
Female	3876	1496 (38.6)	3559	1210 (34.0)	3005	1243 (41.4)	4189	1714 (40.9)	< 0.001
***χ2* and* P*** **value**^**c**^	-		-		-		-		
Age (years)									
45 ~	4408	25.26 (23.77–26.75)	3722	20.76 (19.28–22.24)	2904	23.01 (21.35–24.84)	3719	23.20 (21.65–24.76)	0.12
60 ~	2233	49.25 (46.28–52.22)	2291	41.92 (39.23–44.62)	2192	45.96 (43.07–48.86)	2827	46.71 (44.19–49.23)	0.55
70 ~	1035	61.79 (56.97–66.62)	981	61.79 (56.97–66.62)	1030	62.60 (57.67–67.54)	1458	62.85 (58.74–66.96)	0.54
80 ~	224	77.59 (66.07–89.11)	204	70.12 (58.57–81.68)	236	77.67 (66.29–89.04)	324	73.09 (63.75–82.43)	0.56
***χ2* and * P*** **value**^**c**^	*χ2* = 820.690, *P* < 0.001		*χ2* = 803.653, *P* < 0.001		*χ2* = 743.067, *P* < 0.001		*χ2* = 954.233, *P* < 0.001		
Educational attainment									
Primary school or below	5035	45.96 (44.08–47.85)	4482	41.66 (39.75–43.56)	3902	47.49 (45.32–49.66)	5138	46.74 (44.85–48.63)	< 0.001
Middle and high school	2634	24.73 (22.81–26.64)	2553	20.62 (18.84–22.39)	2292	25.73 (23.59–27.87)	2987	29.42 (27.45–31.38)	< 0.001
College or above	231	17.78 (12.11–23.45)	163	13.85 (8.25–19.48)	168	19.99 (12.87–27.11)	203	15.29 (9.73–20.86)	< 0.001
***χ2* and * P*** **value**^**c**^	*χ2* = 372.670, *P* < 0.001		*χ2* = 351.584, *P* < 0.001		*χ2* = 279.889, *P* < 0.001		*χ2* = 312.573, *P* < 0.001		
Residence									
Rural	4632	40.91 (39.06–42.75)	4274	36.79 (34.97–38.61)	3869	42.43 (40.36 -44.49)	4931	43.62 (41.78–45.47)	< 0.001
Urban	3268	33.88 (31.88–35.88)	2924	28.95 (27.00–30.91)	2493	33.77 (31.49 -36.05)	3397	28.95 (27.00–30.91)	0.002
***χ2* and * P*** **value**^**c**^	*χ2* = 372.670, *P* < 0.001		*χ2* = 351.584, *P* < 0.001		χ2 = 279.889, *P* < 0.001		*χ2* = 312.573, *P* < 0.001		
Geographical location									
East	2976	35.24 (33.10–37.37)	2689	33.21 (31.03–35.38)	2374	36.85 (34.41–39.30)	2964	39.04 (36.79–41.29)	0.33
Central	2451	35.20 (32.86–37.55)	2234	31.55 (29.21–33.88)	1918	35.61 (32.93–38.28)	2661	37.86 (35.53–40.20)	0.55
West	2473	44.38 (41.75–47.00)	2275	36.33 (33.85–38.81)	2070	45.03 (42.13–47.93)	2703	43.04 (40.56–45.51)	0.17
***χ2* and * P*** **value**^**c**^	*χ2* = 6.115, *P* = 0.047		*χ2* = 1.162, *P* = 0.559		*χ2* = 4.892, *P* = 0.087		*χ2* = 0.329, *P* = 0.849		
Medical insurance									
Yes	7382	37.22 (35.83–38.61)	6964	33.29 (31.93–34.64)	5773	37.87 (36.28–39.46)	8113	39.55 (38.18–40.92)	< 0.001
No	518	39.04 (36.79–41.29)	234	44.56 (35.76–53.36)	589	50.98 (45.22–56.73)	215	54.46 (44.29–64.62)	< 0.001
***χ2* and * P*** **value**^**c**^	***χ2*** = 0.648, *P* = 0.047		*χ2* = 12.627, *P* = 0.047		*χ2* = 38.338, *P* = 0.047		*χ2* = 19.294, *P* = 0.047		
Pension(income)									
Yes	830	42.38 (37.95–46.81)	5879	34.34 (32.84–35.84)	4856	38.50 (36.75 -40.25)	7492	39.37 (37.94 -40.79)	< 0.001
No	7070	37.58 (36.15–39.01)	1319	30.63 (27.64–33.62)	1506	41.10 (37.87 -44.34)	836	45.11 (40.56 -49.65)	< 0.001
***χ2* and * P*** **value**^**c**^	*χ2* = 7.343, *P* = 0.007		*χ2* = 6.652, *P* = 0.010		*χ2* = 3.246, *P* = 0.072		*χ2* = 10.258, *P* = 0.001		
**Lifestyle subgroups**									
Internet use									
Yes	253	13.44 (8.92 -17.95)	381	11.64 (8.20–15.09)	417	12.93 (9.39–16.46)	1247	16.73 (14.44–19.01)	0.016
No	7647	38.90 (37.51–40.30)	6817	34.90 (33.49–36.30)	5945	38.50 (39.50–40.50)	7081	44.08 (42.53–45.62)	< 0.001
***χ2* and * P*** **value**^**c**^	*χ2* = 67.347, *P* < 0.001		*χ2* = 88.096, *P* < 0.001		*χ2* = 109.367, *P* < 0.001		*χ2* = 329.669, *P* < 0.001		
Sleep duration									
0 ~ 5 h	1286	49.80 (45.93 -53.68)	1187	42.87 (39.07 -46.67)	1064	47.71 (43.53 -51.89)	1568	49.40 (45.83–52.96)	0.26
5 ~ 7 h	2730	37.24 (34.95 -39.53)	2879	31.19 (29.15 -33.23)	2278	36.68 (34.19 -39.18)	3275	37.11 (35.03–39.20)	0.11
7 ~ 8 h	1549	30.48 (27.73 -33.23)	1435	28.22 (25.47 -30.97)	1132	32.84 (29.48 -36.20)	1428	34.24 (31.21–37.28)	0.004
8 ~ 9 h	1710	35.76 (32.92 -38.59)	1254	32.63 (29.47 -35.79)	1343	37.52 (34.22 -40.82)	1463	39.70 (36.46–42.94)	0.004
9 ~ h	625	42.88 (37.75 -48.01)	443	44.58 (38.36 -50.80)	545	48.89 (43.01 -54.77)	594	78.04 (70.67–85.42)	0.56
***χ2* and * P*** **value**^**c**^	*χ2* = 54.990, *P* < 0.001		*χ2* = 30.901, *P* < 0.001		*χ2* = 29.833, *P* < 0.001		*χ2* = 17.151, *P* < 0.001		
Smoking									
Yes	2527	41.55 (37.09–46.01)	2606	35.98 (31.70–40.26)	1878	40.23 (34.84–45.61)	2411	40.00 (35.68–44.32)	0.88
No	5373	38.18 (36.40–39.96)	4592	34.83 (32.88–36.78)	4484	39.83 (37.91–41.75)	5917	40.15 (38.43–41.87)	< 0.001
***χ2* and * P*** **value**^**c**^	*χ2* = 8.229, *P* = 0.004		*χ2* = 1.001, *P* = *0*.317		*χ2* = 0.100, *P* = 0.752		*χ2* = 0.021, *P* = 0.884		
Alcohol consumption									
Yes	2691	33.47 (30.63–36.32)	2598	28.90 (26.41–31.40)	2393	34.15 (31.17–37.12)	2894	34.30 (31.71–36.90)	0.06
No	5209	41.20 (39.34–43.05)	4600	37.11 (35.23–38.98)	3969	43.03 (40.89–45.17)	5434	43.74 (41.86–45.61)	< 0.001
***χ2* and * P*** **value**^**c**^	*χ2* = 44.585, *P* < 0.001		*χ2* = 49.667, *P* < 0.001		*χ2* = 49.316, *P* < 0.001		*χ2* = 69.713, *P* < 0.001		
**Health status subgroups**									
Multimorbidity									
Yes	2989	39.94 (37.68–42.21)	2993	35.00 (32.88–37.12)	2828	41.30 (38.93–43.67)	4789	42.44 (40.59–44.28)	< 0.001
No	4911	36.95 (35.25–38.65)	4205	32.71 (30.98–34.44)	3534	37.36 (35.33–39.39)	3539	36.42 (34.43–38.41)	0.55
***χ2* and * P*** **value**^**c**^	*χ2* = 7.038, *P* = 0.008		*χ2* = 4.199, *P* = 0.040		*χ2* = 10.292, *P* = 0.001		*χ2* = 30.638, *P* < 0.001		
Self-rated health									
Good	1893	30.64 (28.13–33.15)	1789	29.91 (27.36–32.46)	1524	34.34 (31.35–37.33)	2142	32.43 (30.01–34.84)	0.05
Fair	3938	36.69 (34.80–38.58)	3840	32.63 (30.82–34.43)	3376	38.03 (35.95–40.12)	4027	38.81 (36.88–40.73)	0.001
Poor	2069	47.72 (44.73–50.70)	1569	40.70 (37.50–43.89)	1462	46.83 (43.32–50.34)	2159	49.37 (46.40–52.35)	0.04
***χ2* and *P*** **value**^**c**^	*χ2* = 128.867, *P* < 0.001		*χ2* = 48.156, *P* < 0.001		*χ2* = 53.116, *P* < 0.001		*χ2* = 132.418, *P* < 0.001		
Depression									
No depressive symptoms	4048	32.89 (31.11–34.68)	4812	31.56 (29.97–33.16)	3927	37.94 (35.97–39.90)	4427	37.91 (36.08–39.74)	< 0.001
Depressive symptoms	3479	42.28 (40.10–44.46)	2189	37.43 (34.81–40.05)	2131	39.29 (36.61–41.97)	3334	40.21 (38.03–42.39)	0.17
Depression	373	55.21 (46.90–63.52)	197	47.70 (36.93–58.47)	304	54.28 (45.34–63.22)	567	53.76 (47.11–60.42)	1.00
***χ2* and* P*** **value**^**c**^	*χ2* = 118.990, *P* < 0.001		*χ2* = 40.560, *P* < 0.001		*χ2* = 31.609, *P* < 0.001		*χ2* = 53.125, *P* < 0.001		
BADL disability									
None	6683	34.94 (33.52–36.36)	6100	31.38 (29.97–32.78)	5191	36.52 (34.86–38.17)	6908	36.86 (35.43–38.29)	< 0.001
Mild	656	49.32 (43.94–54.70)	649	41.01 (36.06–45.97)	647	46.87 (41.55–52.19)	767	47.22 (42.22–52.23)	0.98
Severe	561	62.11 (55.57–68.66)	449	53.83 (47.03–60.64)	524	54.97 (48.59–61.34)	653	63.98 (57.70–70.26)	0.37
***χ2* and* P*** **value**^**c**^	*χ2* = 200.074, *P* < 0.001		*χ2* = 112.207, *P* < 0.001		*χ2* = 86.072, *P* < 0.001		*χ2* = 201.980, *P* < 0.001		

*Abbreviation*: *BADL* basic activities of daily living

^a^Prevalence of social isolation in middle-aged and older adults standardized by gender in the combined population

^b^Linear-by-linear association was used to identify trends over time in subgroups

^c^A chi-square test was used to test for differences in the prevalence of social isolation across subgroups by year after standardisation

### Risk factors for social isolation

Table 3 presents the potential risk factors associated with the prevalence of social isolation. Demographic variables (age, gender, educational attainment, type of residence, geographical location, medical insurance and pension) were also included in model 1. The health status variables (multimorbidity, self-rated health, and physical and mental health) based on model 1 were added for model 2; and the lifestyle factors variables (internet use, sleep duration, smoking, and alcohol consumption) based on model 2 were added for model 3.

**Table 3:** Results of generalized estimating equation of the prevalence of social isolation according to sociodemographic characteristics, lifestyle factors, and health outcomes

Variables	Model 1^a^		Model 2^b^		Model 3^c^	
	OR	OR 95% CI	OR	OR 95% CI	OR	OR 95% CI
Intercept	0.268***	0.244–0.294	0.247***	0.223–0.273	0.170***	0.143–0.201
Gender						
Male	1	1	1	1	1	1
Female	1.161***	1.094–1.232	1.116***	1.051 -1.185	1.007***	0.935 -1.084
Age (years)						
45 ~	1	1	1	1	1	1
60 ~	2.471***	2.322–2.629	2.434***	2.285 -2.592	2.377***	2.231 -2.532
70 ~	4.561***	4.213–4.939	4.435***	4.090 -4.809	4.198***	3.869 -4.555
80 ~	8.702***	7.448–10.166	8.267***	7.069 -9.668	7.684***	6.570 -8.987
Educational attainment						
Primary school or below	1	1	1	1	1	1
Middle and high school	0.614***	0.575–0.655	0.640***	0.599 -0.683	0.676***	0.632 -0.722
College and above	0.359***	0.292–0.442	0.391***	0.317 -0.481	0.472***	0.379 -0.586
Residence						
Urban	1	1	1	1	1	1
Rural	1.307***	1.230–1.390	1.250***	1.175 -1.330	1.213***	1.140 -1.290
Geographical location						
East	1	1	1	1	1	1
Central	0.966	0.899–1.038	0.931	0.867 -1.001	0.925*	0.860 -0.994
West	1.276***	1.191–1.367	1.222***	1.140 -1.310	1.199***	1.119 -1.286
Medical insurance						
Yes	1	1	1	1	1	1
No	1.455***	1.302–1.625	1.441***	1.289 -1.612	1.432***	1.281 -1.601
Pension(income)						
Yes	1	1	1	1	1	1
No	1.087***	1.034 -1.143	1.111***	1.057 -1.168	1.132**	1.078–1.189
9 ~ h	1.794***	1.621–1.985	1.278***	1.145 -1.426		
Multimorbidity						
No			1	1	1	1
Yes			0.888***	0.838 -0.942	0.885***	0.834 -0.939
Self-rated health						
Good			1	1	1	1
Fair			1.105**	1.037 -1.178	1.093**	1.025 -1.165
Poor			1.323***	1.220 -1.435	1.272***	1.172 -1.381
Depression						
No depressive symptoms			1	1	1	1
Depressive symptoms			1.096**	1.039 -1.157	1.087**	1.030 -1.148
Depression			1.372***	1.219 -1.546	1.348***	1.196 -1.519
BADL disability						
None			1	1	1	1
Mild			1.121**	1.031 -1.218	1.111*	1.022 -1.207
Severe			1.466***	1.324 -1.623	1.443***	1.304 -1.598
Internet use						
Yes					1	1
No					1.632***	1.441 -1.847
Smoking						
No					1	1
Yes					0.981	0.913 -1.053
Alcohol consumption						
No					1	1
Yes					0.801***	0.752 -0.852
Sleep duration						
7 ~ 8 h					1	1
0 ~ 5 h					1.230***	1.129 -1.340
5 ~ 7 h					1.077*	1.004 -1.156
8 ~ 9 h					1.120**	1.035 -1.211
9 ~ h					1.251***	1.126 -1.391

*Abbreviations*: *OR* odds ratio, *CI* confidence interval

^*^p < 0.05

^**^p < 0.01

^***^p < 0.001

^a^Model 1: Only demographic variables (age, gender, educational attainment, type of residence, geographical location, medical insurance, and pension) were included in the model

^b^Model 2: Adding the health status variables (multimorbidity, self-rated health, and physical and mental health) based on model 1

^c^Model 3: Adding the lifestyle factors variables (internet use, sleep duration, smoking, and alcohol consumption) based on model 2

Participants who were older, had a lower level of educational attainment, lived in a rural region or a western region and had no medical insurance or pension had a higher risk of social isolation than participants in other groups. Notably, the presence of multimorbidity (OR = 0.885, 95% CI = 0.834-0.939) had a protective effect on the prevalence of social isolation, which may be related to the fact that we controlled for too many covariates, and those with multiple chronic conditions may be more likely to be cared for by family and friends when controlling for health status. In addition, participants with poor self-rated physical or mental health were more likely to be socially isolated than those in good health. As expected, participants who did not use the internet (OR = 1.632, 95% CI = 1.441-1.847) were more likely to experience social isolation than those who used the internet. Interestingly, alcohol consumption was a protective factor against social isolation (OR =0.801, 95% CI =0.752-0.852). The effect of smoking also became insignificant after adjustment. Normal sleep duration (7-8 h) was protective compared to sleep disorder, especially for long sleep.

### Age-period-cohort analysis

**Fig. 2:**
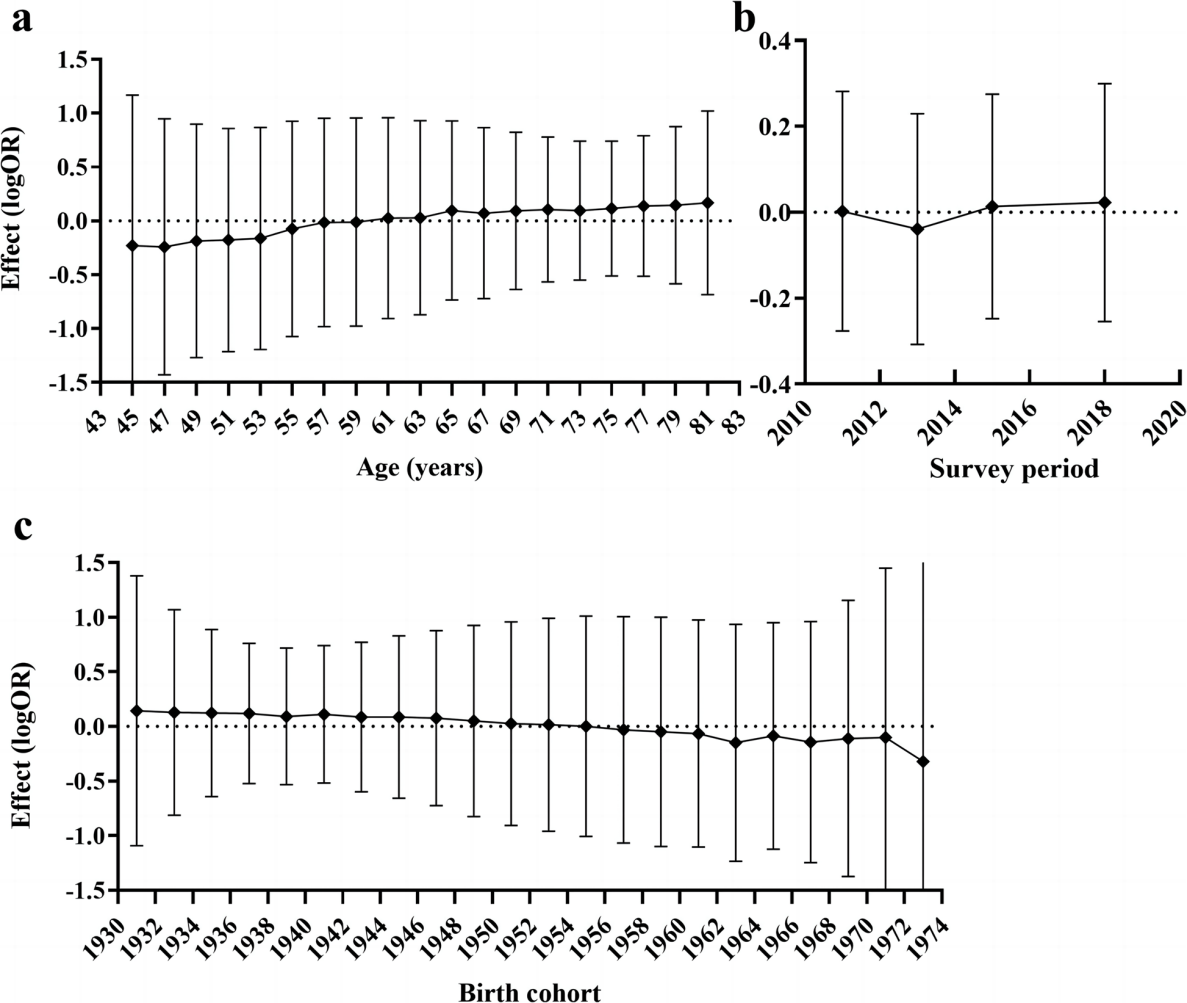
Age-period-cohort analysis of social isolation among middle-aged and older adults in China, 2011—2018

Consistent with the results of the previous analyses, the age-period-cohort analysis also revealed an increasing trend in the prevalence of social isolation, regardless of age, survey period, or birth cohort effects after controlling for the other two effects separately (Fig. 2). The prevalence of social isolation showed a slow upward trend over time among middle-aged and older adults excluding the effects of period, age and birth, especially with increasing age, except for a brief downward trend from 2012 to 2014 (Fig. 2b). This means that the issue of social isolation needs attention, especially in the context of long-term coexistence with COVID-19, whereby the growth trend is likely to be further exacerbated, and measures to reduce social isolation should be actively applied at the societal level, especially among the older age group.

## Discussion

To the best of our knowledge, this is the first study to explore a U-shaped trend in the prevalence of social isolation from 2011 to 2018 and it revealed an increasing trend over time from 2013 to 2018 after adjusting for the covariates among middle-aged and older adults in a nationally representative dataset from China using four cross-sectional surveys over approximately 10 years. In addition, an age-period-cohort analysis confirmed this increasing trend in the prevalence of social isolation over time. A number of issues brought about by an ageing population in the context of rapid urbanization and economic development in China might exacerbate social isolation, such as the ‘rise of living alone’, the ‘epidemic of loneliness’ and dramatic lifestyle changes [54–56].

Similar to the findings of previous studies [47, 57], we also observed a general and gradual increase in social isolation with age. Participants aged over 60 years were more than twice as likely to experience social isolation compared to those aged 45–59 years (41.92%-77.67% vs 20.76%-25.26%). This is comparable to the prevalence of social isolation among older people in studies from other Asian countries [58], although the prevalence of social isolation did not show an upward trend in all age groups during this period. This finding may be explained by the fact that older adults have been exposed to risk factors for isolation for a longer period of time than middle-aged adults due to a greatly reduced social network as a result of old age, such as migration of children, other relatives, and friends, as well as the death or increasing disability of social network members. Some studies have also suggested that the oldest old are more likely to perceive themselves as isolated than the young old, and the source of the greater feelings of isolation and the perceived lack of social support that are more common among the oldest adults should be further investigated [6, 59].

We also confirmed that being female, having a low educational attainment and living in a rural area are risk factors for social isolation, which is consistent with most findings, although contrary evidence also exists [5, 60]. A longitudinal study in China indicated the mechanisms that the moderating effects of education and gender differences on the relationship between social isolation and depression were examined among older adults, and suggested that men with higher education may be more vulnerable to the detrimental effects of social isolation. Policy-based proposals to improve social connectedness are needed [15], especially in rural areas of China where more older adults live alone compared to in urban areas [32]. We found that the prevalence of social isolation varied strongly with the socio-economic factors of interest. The more disadvantaged economically people are in terms of education, medical insurance and pension (income), the higher the prevalence of social isolation. In addition, the increasing trends in social isolation are more pronounced with lower socioeconomic status over time. Social isolation is known to result from a complex interplay of socioeconomic power and inequalities. Our findings support the notion that those who are marginalized are more likely to experience social isolation [61]. It is well known that socio-economic status plays a dominant role in shaping the living conditions and physical environments that provide access and opportunities to develop and maintain social connections. Inequalities in social connectedness begin with education in the early years of life, with lower levels of education being consistently associated with a higher prevalence of social isolation. Therefore, more equal opportunities for a good education may improve social inclusion, as some studies have suggested, to mitigate the subsequent health inequalities of social isolation [5].

Although no increasing trend in the prevalence of social isolation was found among the middle-aged and older adults with the worst mental and physical health, we found that the worse people’s health status, the higher the risk of social isolation. For example, in the depressed group, the prevalence of social isolation over 10 years ranged from 47.70% to 55.21% ; and in the group with severe limitations in activities of daily living, the prevalence was as high as 53.83%-63.98%, with one in two people being socially isolated in this group. When controlling for various confounding factors, the prevalence of social isolation among people in poor health is 1.348-1.466 times higher than among people in good health. Previous studies have confirmed the significant relationship between social isolation and well-being [28–30, 62], which is mutually reinforcing. The main explanatory power of the relationship lies with health being a consistent predictor in the literature. This may be because deteriorating health, particularly in the form of chronic conditions and functional disability, is a major determinant of quality of life in later life [63]. However, the mechanisms by which health and social isolation operate are not clear, e.g. which types of social relationships are more relevant to health and the pathways of causality require further investigation.

In addition, the prevalence of social isolation was found to be three times higher among those who did not use the internet than among those who did, and after controlling for confounders, the risk of social isolation was 1.443 times higher among those who used the internet, which is consistent with previous studies showing that internet use alleviates social isolation [26, 27]. In other words, internet-based contact with the outside world provides social support, participation in activities of interest, and increased self-confidence [59]. However, more than 90% of participants had not used the internet in the previous month, indicating that internet usage was not a common habit among middle-aged and older adults before the COVID-19 pandemic. This may be because it was difficult for some middle-aged and older adults to acquire the basic skills needed to use the internet, and they may not have been able to afford frequent operating system changes [12]. Therefore, skills training in the use of smart devices may be appropriate for this age group. Our findings are similar to a recent cohort study of older adults in Taiwan [25] that found that social isolation was associated with poorer self-reported sleep quality after controlling for demographic, health, cognitive, and depressive factors. In addition, we found that long sleep (9 or more hours) was strongly associated with social isolation, which has only been reported in one previous study [64]. We support the study's point of view that people who are more socially isolated report spending more time in bed in the morning. Interestingly, unlike previous studies [17, 62], we found that smoking was no longer a significant factor influencing social isolation, whereas alcohol consumption was a protective factor against participants’ social isolation after controlling for other variables. A possible reason for this finding is the drinking culture in China, where drinking alcohol is the most common social activity and may strengthen social and business ties [65].

Inevitably, there are some limitations of the current study that need to be highlighted. Firstly, we censored a larger sample of missing variables, which may have made the results of the study more conservative. Furthermore, the trends in social isolation are cross-sectional and only show differences between individuals [66], The within-individual changes in social isolation over time may differ from the cross-sectional trends, and this is a direction for future study [67]. Second, due to the limitations of the secondary database, we only considered individual factors of the study population and ignored the influence of macro-environmental factors, such as policies at the societal level, which will be the focus of the next study. Third, the results of the APC analysis may be biased due to the problem of over-control, and we will continue to focus on the most important influencing factors. In addition, this study examined trends in social isolation among middle-aged and older adults before the COVID-19 pandemic, with the aim of providing insights into the development of policies to address social isolation among older adults during the pandemic.

## Conclusions

In conclusion, we found a U-shaped trend in the prevalence of social isolation from 2011 to 2018 with an increasing trend from 2013 to 2018 among middle-aged and older adults in China, and we provided an in-depth examination of the relationships between social isolation and demographic, lifestyle, and health status. The findings of the study highlight the urgent need for interventions to reduce social isolation including improving sleep quality and internet skills. Disadvantaged groups in terms of age, economic status, and health status should be the focus of such interventions, especially in the era of COVID-19.

### Supplementary Information

**Additional file 1: Table S1.** Variable assignment.

## Data Availability

Researchers may obtain the datasets after sending a data user agreement to the CHARLS team. https://charls.pku.edu.cn/.
