# Supplementary material for: Trends in the prevalence of social isolation among middle and older adults in China from 2011 to 2018: the China Health and Retirement Longitudinal Study
Source: BMC Public Health. 2024 Feb 1;24:339. doi: 10.1186/s12889-024-17734-4 (PMC10832184; doi:10.1186/s12889-024-17734-4)
Supplement: Supplementary file 1 — Additional file 1: Table S1. Variable assignment. [file 12889_2024_17734_MOESM1_ESM.docx]

**Table S1. Variable assignment**

| **Variables** | **Assignment** |
| --- | --- |
| Dependent variables | |
| Social isolation   1. Is the participant cohabiting? 2. Did the participant or their spouse see their parent or parent-in-law in the last month? 3. Did the participant see or contact (including by phone, text message, mail, or e-mail) their child in the last month? 4. Did the participant interact with their friends in the last month? 5. Did the participant engage in any activities (such as social clubs or resident groups, religious groups, or committees) in the last month? | 0 = Non-isolated  1 = Isolated  If three or more of these questions were answered with “no”, a value of 1 was assigned; otherwise, a value of 0 was assigned. |
| Independent variables | |
| Health outcomes | |
| Mental health (depression)  (Centre for Epidemiological Studies Depression Scale) | 0 = No depressive symptoms  1 = Depressive symptoms  2 = Depression |
| Biological health (BADL disability)  (Katz’s basic activities of daily living [BADL] scale) | 0 = none  1 = mild  2 = severe |
| Self-assessed health  (Would you say your health is good, fair, or poor?) | 0 = Good  1= Fair  2 = Poor |
| Multimorbidity  (Are you suffering from two or more chronic diseases?) | 0 = No  1 = Yes |
| Sociodemographic characteristics | |
| Gender | 0 = Male  1 = Female |
| Age  (What is your age this year?) | 0 = 45-59  1 = 60-69  2 = 70-79  4 = ≥80 |
| Educational attainment  (What is your highest qualification?) | 0 = Primary school or below  1 = Middle and high school  2 = College and above |
| Residence  (Is your area of residence rural or urban?) | 0 = Urban  1 = Rural |
| Geographical location  (In which province in China do you reside?) | 0 = East  1 = Central  2 = West |
| Medical insurance  (Do you currently have health insurance?) | 0 = No  1 = Yes |
| Pension  (Do you have currently have a pension?) | 0 = No  1 = Yes |
| Lifestyle variables | |
| Internet use  (In the last month, did you use the internet?) | 0 = No  1 = Yes |
| Sleep duration  (How much sleep do you usually get each night?) | 0=0~5 h  1=5~7 h  2=7~8 h  3=8~9 h  4=9~ h |
| Smoking  (Do you currently smoke?) | 0 = No  1 = Yes |
| Alcohol consumption  (Do you currently consume alcohol?) | 0 = No  1 = Yes |
